# Intradialytic nutrition and quality of life in Chilean older patients in hemodialysis with protein-energy wasting

**DOI:** 10.1007/s11255-021-03077-1

**Published:** 2021-12-03

**Authors:** Mariana Ayala, Margarita Marchant, Cristina Hertz, Gloria Castillo

**Affiliations:** Dialysis Centers NephroCare Chile, Fresenius Medical Care, Duble Almeyda 2915, 7790569 Ñuñoa, Santiago Chile

**Keywords:** Protein energy wasting, Intradialytic oral nutritional supplementation, Quality of life, Nutritional status, Hemodialysis

## Abstract

**Purpose:**

The study assessed the impact of intradialytic oral nutritional supplementation on the quality of life in patients receiving hemodialysis and diagnosed with protein energy wasting.

**Methods:**

A pre-test post-test quasi-experimental study was conducted before and after 3 months of intradialytic oral nutritional supplementation on 109 older hemodialysis patients. We measured before and after 3 months of intradialytic oral nutritional supplementation, the quality of life score, the burden of kidney disease, three quality of life scales and the mental and physical health status using KDQoL-SF™ 1.3, body composition and biochemical parameters of nutritional condition.

**Results:**

The mean age of the patients was 69.4 ± 3.4 years, 59% were male, and the time on dialysis was 63.5 ± 52.6 months. Comparing the baseline with month 3 of intradialytic oral nutritional supplementation, we observed to better quality of life. In contrast to malnutrition, score, specifically increased significantly score of symptoms/problems list related to hemodialysis, sexual function, social and cognitive function, sleep, pain, energy/fatigue and general state of health. Significant changes were also found in nutritional status, energy intake and body composition indicators. After 3 months of intradialytic oral nutritional supplementation, we observed a nutritional status recovery in one or more indicators in 92% of the patients*.*

**Conclusion:**

Our findings indicate that 3 months of intradialysis oral nutritional supplementation improves the components of physical and mental quality of life and nutritional status in older patients receiving hemodialysis diagnosed with loss of protein energy. These results are relevant to improve the experience of patients with protein energy loss receiving hemodialysis.

## Introduction

The term “Protein-Energy Wasting” (PEW) has been suggested to describe a clinical progressive depletion of protein and/or energy stores is often observed which has high prevalence rates (up to 50–75% of patients with CKD stages IV–V) [1]. Protein-energy wasting (PEW) is associated with adverse clinical outcomes including greater morbidity, increased mortality from infection or cardiovascular conditions and impaired quality of life in patients receiving hemodialysis (HD) [2, 3]. Several factors contribute to the development of PEW in HD patients including an inadequate dietary intake, comorbidities, mean dialysis vintage, loss of nutrients during dialysis and metabolic derangement [4, 5].

PEW is most commonly diagnosed using four sets of traditional criteria: biochemical criteria; low body weight, reduced total body fat, or weight loss; a decrease in muscle mass; and low protein or energy intakes [6]. The decline in serum albumin concentration occurring in PEW, which progresses with time on dialysis, is a strong predictor of mortality [7].

Unlike malnutrition, interventions to mitigate the effects of PEW have considered the implementation of physical activity programs together with supplementation strategies aimed at increasing protein and caloric intake, however, in older patients, it is difficult to implement interventions aimed at improving aspects such as muscle strength, the increased protein and caloric intake is a more viable type of intervention [8–10]. Some of the beneficial effects of intradialysis oral nutritional supplementation [ONS] are improvements in serum biomarkers (albumin, prealbumin, and transferrin), weight gain, and increased protein and caloric intake [11–13]. In a prospective observational study, higher serum albumin rates and lower 1-year hospitalization rates were associated with the administration of ONS [14, 15]. Catabolism mitigation has been observed to extend after the end of dialysis. The intake of oral supplements or foods and drinks rich in proteins, during the first hour of dialysis, mitigates the catabolism promoted by the dialysis process [16, 17]. There is evidence that ONS improves nutritional markers, helps maintain a positive amino acid balance during HD [18, 19]. Observational studies have reported a relationship between poor nutritional status and decreased quality of life (QoL) in HD patients, malnourished patients had poorer scores in almost all dimensions of QoL [20–22] and an association between higher malnutrition-inflammation scores and worsening of QoL have been identified. Malnutrition by itself might cause inflammatory status and lead to negative nitrogen balance, weight loss and anorexia, leading to the malnutrition-inflammation complex syndrome. Significant negative correlations were observed between all quality of life aspects and malnutrition-inflammation scores [23–25]

Few studies have evaluated the effect of intradialysis ONS on quality of life in older patients on hemodialysis. Therefore, we investigated whether the administration of ONS for 3 months during the first hour of dialysis would affect the quality of life and nutritional status in HD patients diagnosed with PEW.

## Materials and methods

### Subjects

We prepared a list of 346 patients on hemodialysis regimens 3 times a week from 4 Nephrocare dialysis centers in Santiago of Chile and then each member was assigned with a specific number. From this population, were chosen using random number generator software to 145 patients, of which 121 patients met the inclusion criteria. Twelve patients voluntarily abandoned their participation before the end of the study. A total of 109 participants completed the study, which is the population used for the analysis and presentation of results. Inclusion criteria for participation in the study were: HD for at least 3 months, confirmed diagnosis of PEW, no recent hospitalizations, conscious and able to follow instructions, and intact swallowing ability. Patients with inadequate dialysis [eKt/V < 1.2], presence of cardiac pacemaker [incompatible with BCM measurements], pregnancy, incident dialysis, diagnosis of liver failure, relapsed cancer or de novo, vasculitis, endocarditis, osteomyelitis, diabetics with gastroparesis or symptoms of enteropathy, major surgery in the last 6 months, nutritional supplementation or complete enteral nutrition in the last 3 months, were excluded from the study.

### Study design

This was a pre-test post-test quasi-experimental study design; each patient serves as her own control. The impact on quality of life and nutritional status of 3 months of intradialysis ONS in HD patients diagnosed with PEW was evaluated. As part of routine care, all patients received nutritional counseling from a nutritionist during the HD session. The intake of macro- and micronutrients of 3 days [dialysis day, day without dialysis and weekend day] was evaluated. The determination of the dietary pattern considered the frequency of weekly food consumption to avoid underestimating the caloric and energy intake in the dietary pattern.

**Fig. 1:**
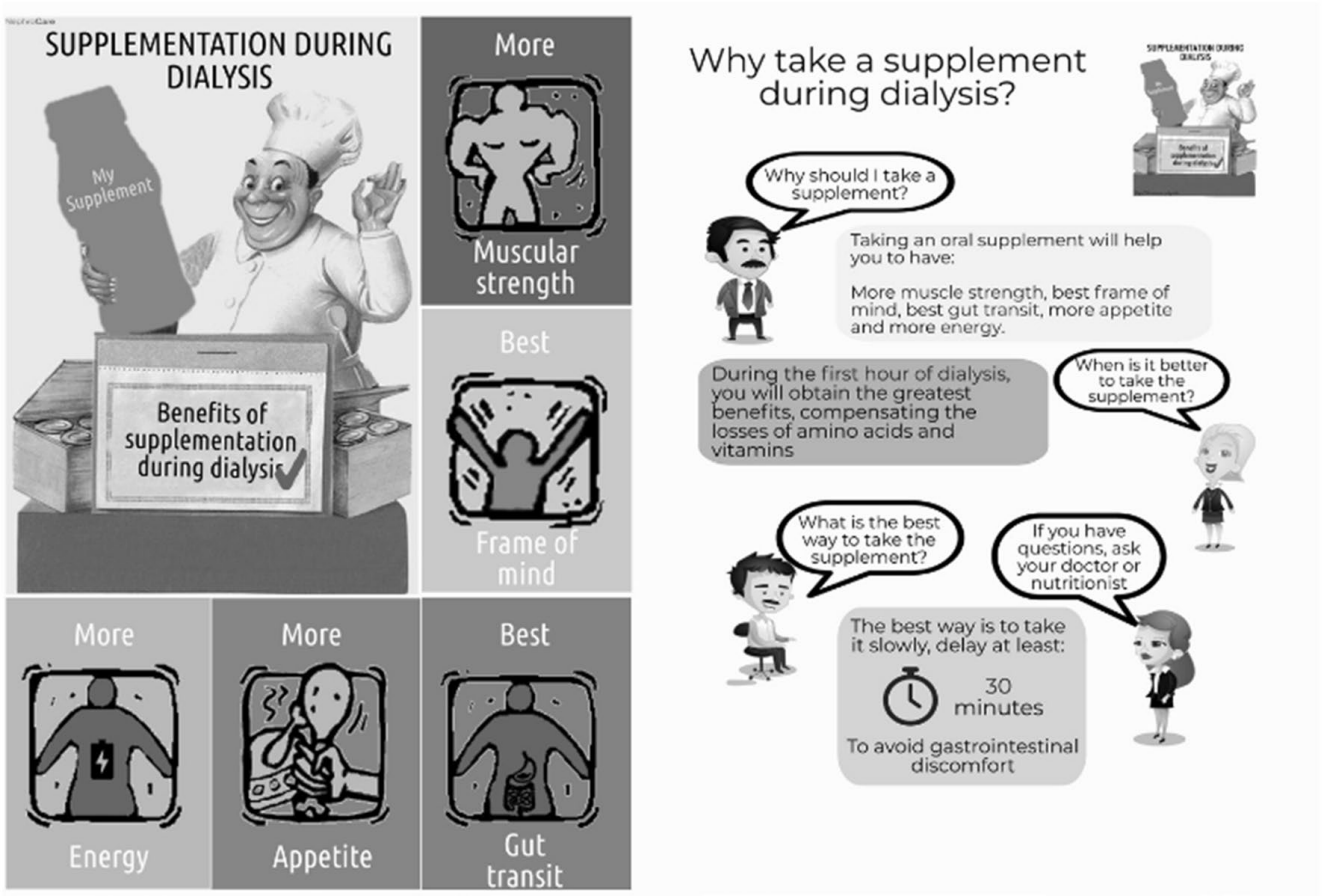
Benefits of intradialytic ONS. Benefits of oral nutritional supplementation during dialysis and indications on how to ingest the supplement, based on the main questions of hemodialysis patients

The nutritional information was delivered focused on the benefits of intradialytic ONS and the patients’ frequent questions about supplementation (Fig. 1).

Blood samples were taken at baseline and 3 months after supplementation immediately prior to the dialysis session.

We evaluate at the beginning and end of the study; Biochemical and body composition indicators. Using KDQoL-SF™ 1.3, quality of life score, burden of kidney disease, three quality of life scales, and mental and physical health status were assessed.

This study was conducted in accordance with the principles of the Declaration of Helsinki. The protocol was approved by the local Ethics Committee. All participants received written and verbal information on the objectives and procedures of the study and provided a signed consent form.

### Biochemical and body composition parameters

Serum blood samples were taken for biochemical analysis and indicators of nutritional status at the beginning of the study and at month 3, immediately before HD. The samples were sent for analysis to a central laboratory at each dialysis center.

Data on body composition and hydration status were collected before the HD session, at the beginning and at the end of the study, by means of bioimpedance spectroscopic analysis at 50 frequencies (5 kHz–1000 MHz), with the Body Composition Monitor system^®^(BCM). (Fresenius Medical Care, Deutschland GmbH, Germany^®^).

Measurements were taken using four conventional electrodes placed on the patient in the supine position, approximately 30 min before each HD. The extracellular and intracellular fluid volumes and total body water were calculated from the fluid model. These fluid volumes were then used to determine the fluid overload, expressed as OH value [26]. Dry weight BCM was calculated by pre-weight minus OH (kg).

The nutritional status was evaluated by the following measures: albumin, normalized C-reactive protein (CRP); percentage of involuntary weight loss and body composition parameters (dry weight, BMI, lean tissue mass, adipose tissue mass, fat tissue mass, lean tissue index, fat tissue index, relative lean tissue mass, relative mass of fatty tissue). The diagnosis of PEW followed the nomenclature and diagnostic criteria proposed by Fouque (Table 1). Biochemical and body composition data of the patients obtained from the electronic EuCliD registry (European Clinical Database) [27] were used for the diagnosis of PEW.

**Table 1:** Criteria for the clinical diagnosis of PEW in study population (*n* = 109) according to Fouque 2008

Criteria
Serum chemistry
Serum albumin < 3.8 g per 100 ml
Body mass
BMI < 23 kg/m^2^
Unintentional weight loss over time: 5% over 3 months
Total body fat percentage < 10%
Muscle mass
Muscle wasting: reduced muscle mass 5% over 3 months
Dietary intake
Unintentional low dietary protein intake < 0.80 g/kg/day for at least 2 months for dialysis patients^a^
Unintentional low dietary energy intake < 25 kcal kg/day for at least 2 months

Criteria for diagnosis of PEW: At least three out of the four listed categories (and at least one test in each of the selected categories) must be satisfied for the diagnosis of kidney disease-related PEW

^a^Was assessed by dietary records and protein intake by calculation of normalized protein equivalent of total nitrogen appearance (nPNA or nPCR) as determined by urea kinetic measurements

### Intradialytic ONS

During the first hour of HD for a period of 3 months, each patient received 200 ml of an oral supplement, consisting of 400 kcal, 20 g of protein, 15.6 g of lipids and 45 g of carbohydrates, 3 g of fiber, 12 mg/g phosphorus: protein ratio (Fresubin 2 kcal Drink®; Fresenius Kabi). During the supplementation time, no gastrointestinal symptom was observed.

### Assessment of quality of life

The KDQoL-SF ™ 1.3 form digital version (QR code) was used to measure the specific quality of life score for kidney disease [28]. The form was administered before the HD session at the beginning and at the third month of supplementation and was used to evaluate: (1) list of symptoms/problems, effects of kidney disease, burden of kidney disease, work status, cognitive function, quality of social interaction, sexual function, family and friend support, sleep and dialysis staff support; (2) general health, patient satisfaction; (3) physical function, role of physical limitations, pain, general health, emotional well-being, role of emotional limitations, social interaction, energy/fatigue; (4) composite of physical health and composite of mental health. Using the scoring manual, the following scoring ranges were used to classify each component of quality of life: (i) completely inappropriate [score 0–20]; (ii) inappropriate [score 20–40]; (iii) low [score 40–60]; (iv) adequate [score 60–80]; (v) completely appropriate (score 80–100) [29].

### Statistical analysis

The main parameter of this study is quality of life and the second parameter is nutritional status, evaluated as changes in the quality of life score and nutritional indicators (body composition and biochemicals), respectively, from baseline to month 3 The quality of life score is shown descriptively as the mean of the percentage score and the standard error with a score of 100 indicating an improvement in quality of life. Improvement in nutritional status was defined as a significant change from baseline measurements of any of the following parameters: nPCR, serum albumin, BMI, and percentage of involuntary weight loss.

The data distribution was analyzed using the Kolmogorov–Smirnov test. Comparisons between variables were made using the paired *T* test or the Mann–Whitney *U* test, depending on the distribution of the data. The sample size was based on albumin concentrations, considering a maximum variation of 5% for the detection of a mean difference in albumin levels of ≥ 2.5 g/L with a probability of 95% and *α* = 0.05. The sample size considered in this study was 109 HD patients. All statistical tests were performed with the SPSS 17.0 statistical package [SPSS, Inc., Chicago, IL and USA]. Statistical significance was defined as *p* < 0.05. Data are shown as the mean and standard error or deviation.

## Results

### Patient baseline characteristics

The study population comprised 109 participants diagnosed with PEW. 59% males and 41% females participated, the mean age was 69.4 ± 3.4 years (75.2% older than 66 years), 35.8% had type 2 diabetes and the mean duration of HD was 63.5 ± 52.6 months, the adequacy of OCMKt/V and eKt/V dialysis remained in the optimal range throughout the study. The main causes of kidney failure were glomerular abnormalities in type 2 diabetes (*n* = 39) hypertensive kidney disease (*n* = 18), polycystic kidney disease (*n* = 4), and unknown cause (*n* = 48) (Table 2).

**Table 2:** Demographic characteristics of the study population (*N* = 109)

Characteristic	
Age, years (mean ± sd)	69.4 ± 3.4
Male *n* (%)	64 (59)
Dialysis vintage, mean (minimum—maximum; months)	63.5 ± 52.6 (4–216)
Type 2 diabetes, *n* (%)	39(35.8)
OCM Kt/V	1.72 ± 0.3
eKt/V	1.52 ± 0.2
Main causes of renal failure (%)	
Type 2 diabetes	35.8
Hypertensive renal disease	16.5
Polycystic kidney disease	3.7
Unknown cause	44
Vascular access type (%)	
Arteriovenous fistula AVF	72.4
Arteriovenous graft AG	1.7
Peripheral venous catheter PVC	21.6
Tunneled catheter TC	4.3

*OCM Kt/V* online clearance monitor of delivered dose of dialysis

*eKt/V* equilibrated Kt/V

### Dietary records

A significant increase in the intake of calorics, macronutrients and micronutrients was observed. When comparing the intake between the beginning and after 3 months of supplementation, a significant increase was observed in: total energy consumption (1626.2 ± 328.5 vs. 1856.4 ± 275.4 kcal/day), protein (61.4 ± 12.3 vs. 67.5 ± 9.7 g/day), lipids (50.8 ± 16 vs. 56.4 ± 9.9 g/day) and carbohydrates (230.8 ± 45.6 vs. 269.6 ± 42.9 g/day). A significant increase in phosphorus intake of 214.5 mg/day (833.1 ± 209.4 vs. 1047.7 ± 300.8 mg/day) was associated with the net increase in protein intake provided by ONS. In contrast, blood phosphorus levels decreased significantly [see biochemical parameters section below]. Significant increases were also found in dietary potassium intake (1919.5 ± 726.5 vs. 2405.7 ± 535.3 mg/day) and calcium (392.2 ± 178.4 vs. 525.2 ± 95.3 mg/day). Sodium intake (2417.2 ± 977.4 vs. 2345.6 ± 609.8 mg/day) remained without significant changes compared to the initial value and after 3 months of supplementation.

### Biochemical and body composition parameters

Significant changes were observed in the indicators of nutritional status and body composition. An improvement in one or more indicators was observed in 92% of the patients. A worsening of the nutritional status was observed in 8% of patients, comparing the baseline and month 3 of supplementation. Serum bicarbonate levels at the beginning and at the end of the study remained in an adequate range (22–26 mmol/L), no metabolic acidosis was observed. However, a significant decrease in serum bicarbonate levels was observed when comparing before and 3 months after supplementation (Table 3).

**Table 3:** Biochemical and body composition parameters of patients in HD with PEW before and after supplementation with intradialytic ONS (*n* = 109)

Parameters	Intradialytic ONS		
Biochemical	Before mean ± SD	After Mean ± SD	*P*
Albumin (g/dl)	3.72 ± 0.31	3.79 ± 0.27	*0.036*
nPCR^a^ (g/kg/d)	1.07 ± 0.27	1.08 ± 0.26	*0.301*
Phosphorus (mg/dl)	4.62 ± 1.64	4.22 ± 1.56	*0.001*
Creatinine (mg/dl)	7.88 ± 2.25	7.47 ± 2.28	*0.210*
Potassium (mEq/L)	5.06 ± 0.67	5.03 ± 0.53	*0.350*
Bicarbonato (mmol/L)	23.35 ± 2.61	21.92 ± 2.17	*0.000*
Hemoglobin (g/dl)	10.78 ± 1.83	11.13 ± 1.91	0.040
Body composition			
Dry weight (Kg)	62.23 ± 12.14	62.96 ± 12.84	*0.003*
Unintentional weight loss in 3 months (%)	0.84 ± 3.21	-1.22 ± 3.46	*0.005*
Body Mass Index (kg/m^2^)	24.03 ± 4.05	24.32 ± 4.32	*0.000*
Lean tissue mass (kg)	30.61 ± 8.64	29.54 ± 8.41	*0.110*
Adipose tissue mass (kg)	30.69 ± 12.09	33.1 ± 12.89	*0.000*
Fat mass (kg)	22.57 ± 8.88	24.32 ± 9.47	*0.001*
Lean Tissue Index (LTI kg/m^2^)	11.72 ± 2.67	11.30 ± 2.58	*0.270*
Fatty Tissue Index (FTI kg/m^2^)	11.93 ± 4.75	12.88 ± 5.07	*0.046*
Relative Lean tissue mass (%)	48.66 ± 13.43	46.34 ± 13.33	*0.115*
Relative Adipose tissue mass (%)	34.59 ± 10.32	36.59 ± 10.13	*0.001*
Relative Weekly Overhydration (%)	16.01 ± 10.39	11.83 ± 10.51	*0.000*

Mann–Whitney *U* were used to compare data at baseline and 3 months of supplementation with intradialytic ONS. (*P* < 0.05) was considered significant

^*a*^*nPCR* Normalized Protein Catabolic Rate

### Quality of life

All patients (*N* = 109) completed a KDQoL-SF form at the beginning of the study and at the third month of supplementation.

**Fig. 2:**
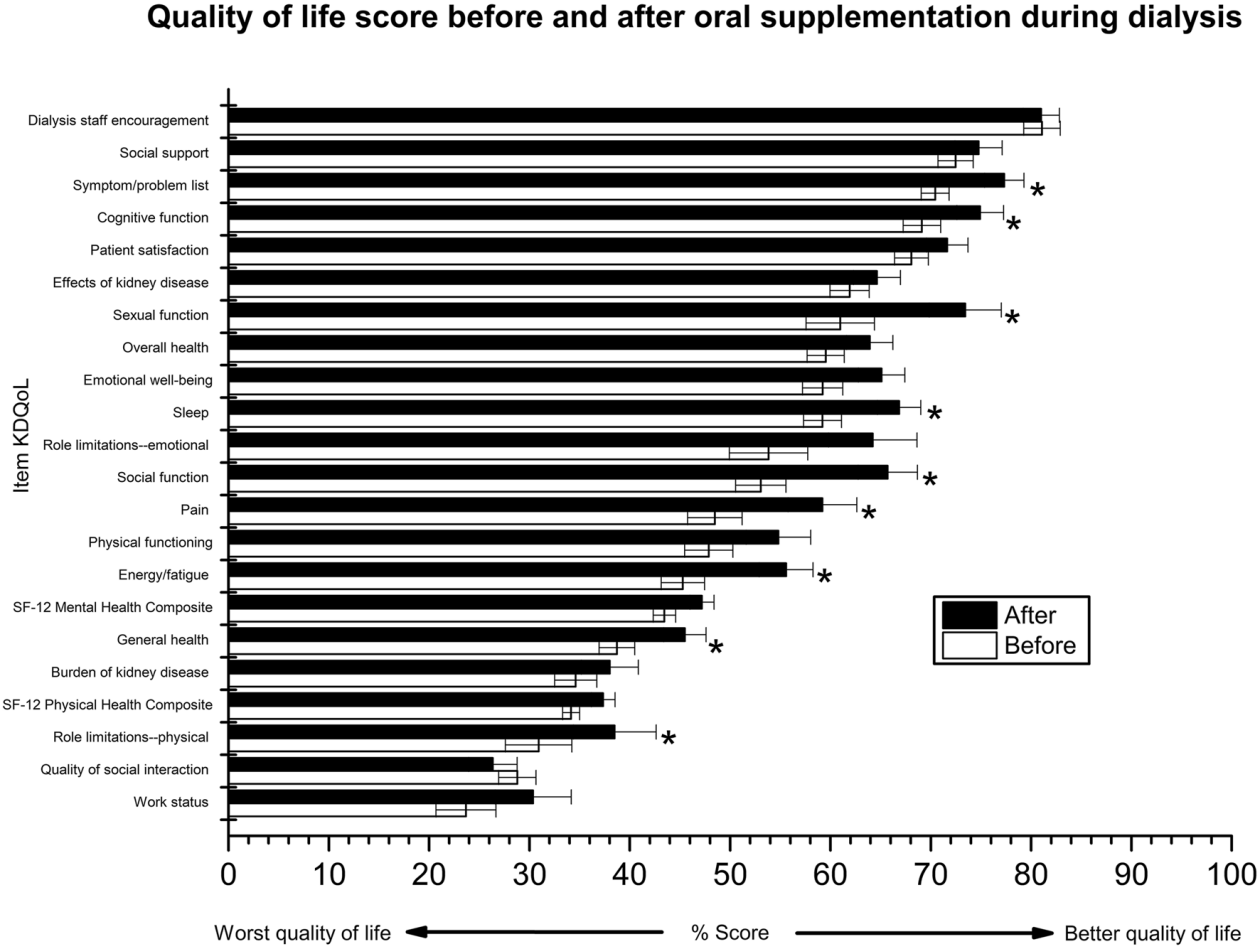
Mean quality of life score at baseline and after 3 months of intradialytic ONS in HD patients diagnosed with PEW. The QoL data are shown descriptively as the mean and standard error of score [0–100], whereby 100 indicates a better QoL. *T* tests for paired samples or Mann–Whitney *U* were used to compare data at baseline and 3 months. *Significantly different versus baseline status (*P* < 0.05)

A significant improvement was observed in the physical and mental components of the quality of life when comparing the baseline and month 3 of supplementation. The scores for the following components increased significantly; symptoms/problems, cognitive, sexual and social function, sleep, pain, energy/fatigue, general health and physical limitations, in particular sexual function, general health, emotional well-being, sleep, functional limitations: emotional and social function. The following components of quality of life increased significantly from the low range (40–60%) to the adequate range (60–80%); sexual function, sleep and social function (Fig. 2).

### Implications for clinical practice

These data contribute to the development of strategies to reduce morbidity and mortality. Treatment and prevention of PEW with intradialysis ONS can help reduce some physical and mental limitations in HD patients. The evaluation of the quality of life is useful to design intervention strategies that improve the clinical outcome, adherence and reduce pain in patients with high metabolic deterioration.

## Discussion

The older hemodialysis patient population is growing rapidly across the world. Recently, attention has focused on the development of PEW. In older patients, PEW is an underlying condition between sarcopenia and frailty, which is mainly caused by a decrease in food intake and increased catabolism. Various treatments have been designed to mitigate the symptoms of PEW [30]. Our study indicates a benefit of intradialytic ONS in both quality of life and nutritional outcomes in older patients without reversing PEW. A large part of the patients had a BMI over 23 kg/m^2^. It is known that PEW is common in overweight and obese patients [8, 31], for this reason, for the diagnosis of PEW, at least three indicators of a set of indicators were considered according to Fouque 2008.

Even after a brief administration of ONS for 3 months, a significant improvement was observed in nine of the 22 components of the QOL, both physical and mental.

Patients receiving HD often complain of difficulty falling sleep and staying sleep, and complaints of fatigue are common in this population. Fatigue induces a protein and caloric intake below 80% of the recommended levels for a day of dialysis, which negatively affects nutritional status [32–34].

Intradialytic ONS significantly increased energy/fatigue and overall health scores, while increasing protein and caloric intake. Tiredness can hamper the ability to prepare proper meals and perform other activities of daily living. The reduction in fatigue is related to a better disposition of patients to eat post-dialysis food.

Lower scores on the physical and mental component of quality of life are known to be associated with an increased risk of death and hospitalization in the next 2 years [22, 35]. Similar to our study, Scott et al. found a significant improvement in scores on the physical-role component using ONS. Our results seem to suggest a positive effect of intradialysis ONS on the physical and mental components of quality of life, but did not indicate significant changes in muscle. A finding also reported by similar studies has shown that muscle protein stores are not solely determined by nutrient intake [36]. Physical activity programs combined with ONS could be an option for muscle strengthening, an effect reported after physical exercise in malnourished young adult patients [18]. In older patients, it is difficult to implement physical activity due to frailty and morbidity, however, in the first stage, increasing protein and caloric intake is a more viable type of intervention and then, in the second stage, a physical activity program could be implemented focused on regaining muscle strength.

Epidemiological studies have shown an increased risk of mortality among dialysis patients affected by sleep disturbances [37, 38]. In our study, the sleep score increased significantly in month 3 of supplementation compared to baseline, going from low (< 60%) to adequate (> 60%), together with the social function score increased significantly, which could consequently affect general well-being and mental health status [21].

Simultaneously with a better QoL score, we observed better nutritional status and a significant increase in albumin at month 3 after supplementation. These changes could be associated with a 29% reduction in the risk of mortality [39, 40]. Despite the increase in protein intake, a significant decrease in serum phosphorus levels was observed, reducing the risk of hyperphosphatemia, a risk factor for mortality in HD patients [41]. The decrease in serum phosphorus levels could be related to a better disposition to follow nutritional medical indications, according to the dietary registry and nutritional anamnesis. In addition, a decrease in serum bicarbonate levels is observed when compared before and after supplementation, related to the increase in protein caloric intake [47].

Most of the patients in our study experienced an improvement in at least one nutritional indicator. However, a worsening of nutritional status was observed in a small group (8%) of patients. This group of patients who did not respond to the intradialysis ONS intervention had indicators of nutritional status such as reserving muscle mass below that recommended at the beginning of the study and did not achieve significant weight gain at the end of the supplementation period.

In this study, an increase in adipose tissue mass, fat mass, fat tissue index and fat tissue index associated with intradialysis ONS was also observed, which has recently been known to contribute to reducing mortality in this group of patients, such as it has been observed in several studies [41, 43, 44]. Along with changes in body composition, we observed a better control of fluid intake, reflected in a significant decrease in OH%. Previous studies have linked higher protein and calorie intake with better OH status [45].

The inclusion of a control group that did not receive ONS would help to better isolate the effect of supplementation on nutritional and quality of life measures. For ethical reasons, a control group could not be included in this study. We did not include identification of psychosocial status (e.g., depression symptoms and anxiety trait), which can potentially affect quality of life, at the beginning of the study. However, the evaluation of these conditions would have required the use of additional specific surveys that would require more time from the patient to complete the questionnaire.

Another limitation of the study is that we were unable to measure C-reactive protein (CRP), an inflammatory biomarker. However, we found an increase in serum albumin, which could be explained by an increase in energy and protein consumption after the intervention [46].

More evidence is needed from randomized controlled trials to provide information on how nutritional support in older HD patients with PEW improves quality of life and nutritional status.

## Conclusions

Our findings indicate that ONS during the first hour of dialysis improves the components of nutritional status and quality of life in older dialysis patients diagnosed with PEW. Improving physical and mental capabilities can help maintain autonomy and improve the experience of patients with hemodialysis treatment. These results are clinically relevant and must be confirmed with long-term supplementation studies in this population.
